# Results of a Phase I-II Study on Laser Therapy for Vaginal Side Effects after Radiotherapy for Cancer of Uterine Cervix or Endometrium

**DOI:** 10.3390/cancers12061639

**Published:** 2020-06-21

**Authors:** Anna Myriam Perrone, Marco Tesei, Martina Ferioli, Francesca De Terlizzi, Anna Nunzia Della Gatta, Safia Boussedra, Giulia Dondi, Andrea Galuppi, Alessio Giuseppe Morganti, Pierandrea De Iaco

**Affiliations:** 1 Gynecologic Oncology Unit, Sant’Orsola-Malpighi Hospital, 40138 Bologna, Italy; marco.tesei2@gmail.com (M.T.); anna.ndg1@gmail.com (A.N.D.G.); safia.boussedra@studio.unibo.it (S.B.); giulia.dondi@gmail.com (G.D.); pierandrea.deiaco@unibo.it (P.D.I.); 2 Centro di Studio e Ricerca delle Neoplasie Ginecologiche (CSR), University of Bologna, 40138 Bologna, Italy; andrea.galuppi@aosp.bo.it (A.G.); amorganti60@gmail.com (A.G.M.); 3 Radiotherapy Unit, Sant’Orsola-Malpighi Hospital, 40138 Bologna, Italy; m.ferioli88@gmail.com; 4 Scientific & Medical Department, IGEA S.p.A., Via Parmenide 10/A, 41012 Carpi (Mo), Italy; f.deterlizzi@igeamedical.com

**Keywords:** cervical cancer, endometrial cancer, vaginal fibrosis, sexual function, radiotherapy, brachytherapy, laser therapy

## Abstract

Women who have previously received radiotherapy (RT) for gynecologic cancer often suffer from vaginal fibrosis and stenosis. The success of “non-ablative” laser therapy for postmenopausal vaginal atrophy has led to the idea of testing the laser in patients submitted to RT. In this prospective observational study, we selected patients who underwent pelvic RT followed by vaginal laser treatment. We scheduled three treatment sessions (at T0–T1–T2) and three controls (at T1–T2–T3) one month apart. The follow-up (at T4) was carried out six months after the last treatment. Vaginal Health Index (VHI) and vaginal length were evaluated. Sexual function was assessed through Female Sexual Function Index (FSFI). Overall, 43 patients with severe vaginal shortening, atrophy and stenosis was enrolled and treated with intravaginal non-ablative CO_2_ laser. We observed a progressive increase in vaginal length of 9% (*p* = 0.03) at T2 and 28% (*p* < 0.0001) at T3; effects were maintained at T4 (*p* < 0.0001). After the first application VHI showed a significant improvement of 57% at T3 (*p* < 0.0001). The results were maintained at T4 (*p* < 0.0001). No changes were found in FSFI. All procedures were well tolerated. In conclusion, laser therapy improved vaginal length and VHI in women undergoing pelvic RT; prospective studies are needed.

## 1. Introduction

Radiotherapy (RT) represents a cornerstone in the treatment of gynecological tumors and, in cases of advanced cervical cancers, it is the only available therapy with curative intent [1,2,3,4,5,6]. However, some side effects affecting sexual function are well known. The impact of external beam RT (EBRT) and brachytherapy (BT) on reproductive organs and sexual activity depends on several factors: age, irradiated volumes, dose and fractionation and treatment technique and duration [7,8,9]. Although dysfunctions of the genital organs after RT may be temporary, recovery is often uncertain—and in some cases—the damage may be permanent. RT, alone or combined with other cancer treatments such as surgery and chemotherapy, induces early menopause and worsens the genitourinary symptoms of menopause in women of different ages, including older patients [10]. RT on the genitals may cause vaginal damage that starts with inflammation of the mucosa and sub-mucosa leading to the development of ulceration, necrosis and fibrosis. The vaginal walls become atrophic, inelastic, narrow, short and lastly stenotic which may make sexual intercourse painful or uncomfortable and, in cases of complete stenosis, impossible [11,12,13,14]. Several studies show that late side effects after RT for gynecological cancer decrease QoL and sexual functioning [10,15,16]. Early assessment and identification of the invalidating symptoms is an important part of care in cancer surviving women. Local hormone therapy and the use of the vaginal dilator, in combination with psychoeducational support, are generally recommended. However, existing literature lacks randomized controlled trials on this topic [15,17,18,19]. The outcomes of these treatments are poor because pelvic RT locally reduces vaginal hormone receptors making the administration of hormones less effective. Moreover, vaginal dilators and psychoeducational support, although useful, can cause pain and discomfort which can result in poor compliance [20,21,22,23].

Intravaginal laser therapy is a new non-pharmacological treatment successfully proposed especially for the management of symptoms produced by menopausal genital atrophy. Due to the satisfactory efficacy of this treatment, the term “vaginal rejuvenation” has been proposed by some authors [24,25,26,27,28].

Other studies reported achievement of rejuvenation of vaginal tissue and improved satisfaction of sexual function by increasing lubrication during intercourse using different types of lasers (erbium laser or CO_2_ laser) [29,30]. In particular, the CO_2_ laser system can induce acute thermo-ablative or non-ablative effect, followed by fibroblast proliferation and stimulation of the synthesis of new mature components of collagen and extracellular matrix that improve the hydration and elasticity of the vaginal walls [27].

Based on these results—and considering the lack of effective therapies to reduce fibrosis and restore sexual activity in women submitted to pelvic RT for gynecological tumors—we decided to test intravaginal laser therapy using a non-ablative laser system in this setting of patients.

In the literature, vaginal improvement in response to laser therapy has been evaluated mainly using physician reported symptoms scales such as the vaginal health index (VHI) [31] and questionnaires as the female sexual function index (FSFI) [32]. These tools have been mostly applied in menopausal patients with vaginal atrophy [33,34]; however, no studies investigating vaginal toxicity after RT have been reported.

The main objectives of the present study were to assess vaginal functional changes by means of VHI score and vaginal length after RT and laser therapy. The secondary objective was to evaluate variations of the sexual function through FSFI.

## 2. Materials and Methods

### 2.1. Study Design, Inclusion Criteria and Data Collection

This is a phase I–II prospective clinical study on the feasibility, safety and efficacy of laser therapy in women with vaginal toxicity after radiotherapy for gynecological cancer. The study was conducted between November 2017 and December 2018 at the gynecological oncology unit of the Policlinico di Sant’Orsola, Bologna, Regional Center of reference for gynecological malignancies. The approval form of the Area Vasta Emilia Centro Independent Ethical Committee (CE-AVEC) number 401/2018/Oss/AOUBo was obtained and the participants signed an informed consent form.

Women with genital cancer that were treated with RT and that underwent intravaginal laser therapy were included in this analysis. Patients’ data were collected using an electronic database. Data recorded included patients and treatments characteristics (age, BMI, comorbidity, gynecological and obstetric anamnesis, histological type and tumor stage, type of surgical therapy, RT dose and technique and chemotherapy), VHI parameters (vaginal elasticity, vaginal secretions, pH, epithelial mucous membrane and vaginal hydration) and sexual function (FSFI score).

In our hospital, patients with gynecological neoplasms that are treated with RT are followed up with regularly scheduled checkups at the gynecologic oncology outpatient clinic where they are examined together with a radiation oncologist to allow multidisciplinary care and reduce the duplication of follow-up visits. Among these patients, laser therapy was proposed to a group of women under 65 years of age and treated with EBRT and/or BT for pelvic tumors, who complained of vaginal discomfort with associated stenosis and fibrosis and difficulty in sexual intercourse, but with the desire to improve vaginal health.

Exclusion criteria were active genital infection or recurrent urinary tract infection, less than six months since the last cancer treatment, diagnosis or suspicion of disease recurrence.

### 2.2. Laser Treatment

**Figure 1:**
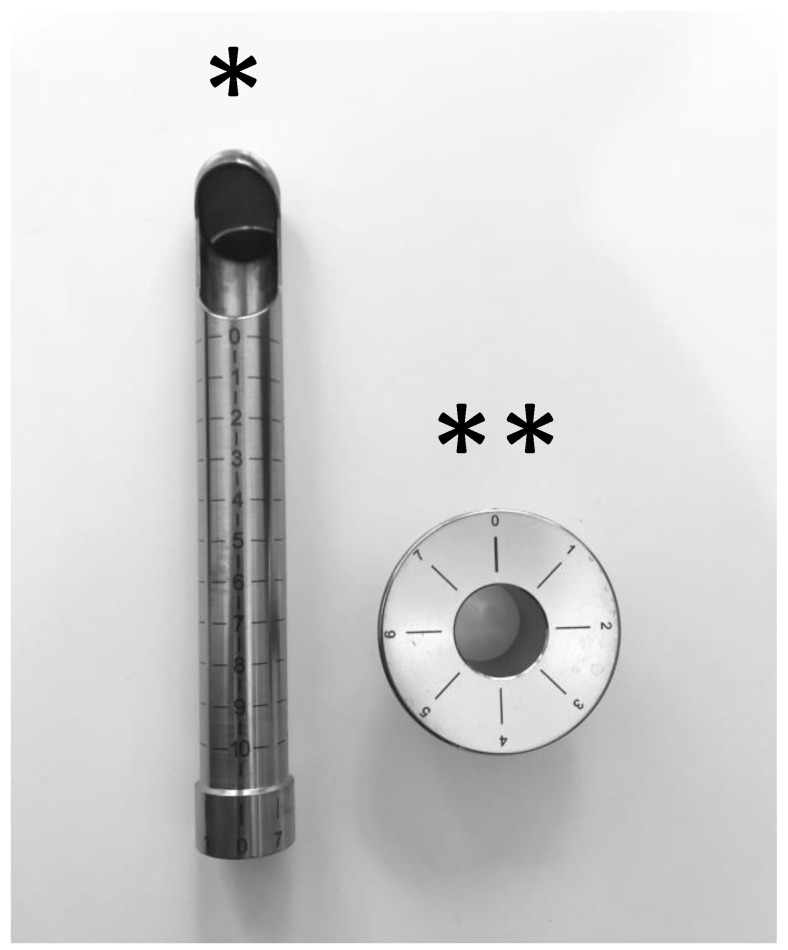
The figure represents the vaginal device of the laser. * Vaginal probe, ** graduated guide.

Laser treatment was performed using an intravaginal non-ablative CO_2_ laser (V-Lase^®^ Lasering, Modena, Italy); this device is composed by a metal probe that is inserted into the vagina through a graduated guide positioned at the level of the vaginal introitus (Figure 1).

The protocol was as follows: after positioning the speculum, both pH and vaginal length were measured and the vaginal secretions were removed. The speculum was replaced with the graduated vaginal guide and then the intravaginal probe was introduced up to the vaginal dome or uterine neck based on any previous surgery. V-Lase^®^ is a “non-ablative” laser, it has a “cylindrical” laser beam with a 10 mm diameter (which therefore affects the “target” on a circular surface of 0.785 cm^2^). The laser was set as follows: pulse duration of 1.5 ms with an interval of 1.5 ms between pulses; emission time of the laser sequences 150 ms; average power 13 W. With this setting the energy density was 1.24 J/cm^2^.

The intravaginal probe progressively distributes the pulses first, in a circumferential direction and then in a longitudinal direction in order to cover the entire vagina.

Two steps are needed to treat the vaginal walls. During the first step, after the first release of energy, the probe is rotated 45° clockwise 8 times, releasing energy at each rotation while remaining at the same depth. During the second step, after the 8 pulses, the procedure is repeated by retracting the probe by one centimeter and replicating the process until the entire surface of the vagina is treated. Overall, the treatment plan included three laser sessions: at baseline (T0), at thirty (T1) and at sixty days after baseline (T2). The procedure did not require anesthesia nor support therapy.

The intensity of perceived pain during the procedure was recorded using a visual analog scale (VAS) (range 0–10, 0 = total absence of the symptom and 10 = the worst possible symptom) [35]. Furthermore, adverse effects during and after treatment and the acceptability of the procedure (low, medium, high) were recorded in the medical record at each visit.

### 2.3. Genital Parameters

The assessment of vaginal functional changes after RT and laser therapy was evaluated using the VHI Score (Tables S1–S4) (range 5–25) based on five parameters: vaginal elasticity, vaginal secretions, pH, epithelial mucous membrane and vaginal hydration. The final score defines the degree of atrophy in the genitourinary tract and it is achieved by assigning a single score to each parameter. Each parameter was graded from 1 to 5, with a total score ≤15 defining an atrophic condition [31]. Sexual function was evaluated using FSFI (Supplementary materials) which is composed by six domains for 19 questions; 17 of 19 include a 5-point scale and 2 of 19, a 6-point scale. The total score is obtained from the sum of the scores of all the domains and ranges from 2 to 36: desire/libido (2 questions), arousal (4 questions), vaginal lubrication (4 questions), orgasm (3 questions), global satisfaction or “global quality of life” (3 questions) and pain (3 questions). A total score >26.5 is commonly used as a cutoff score to distinguish women with normal sexual function from those with sexual dysfunction [36].

**Figure 2:**
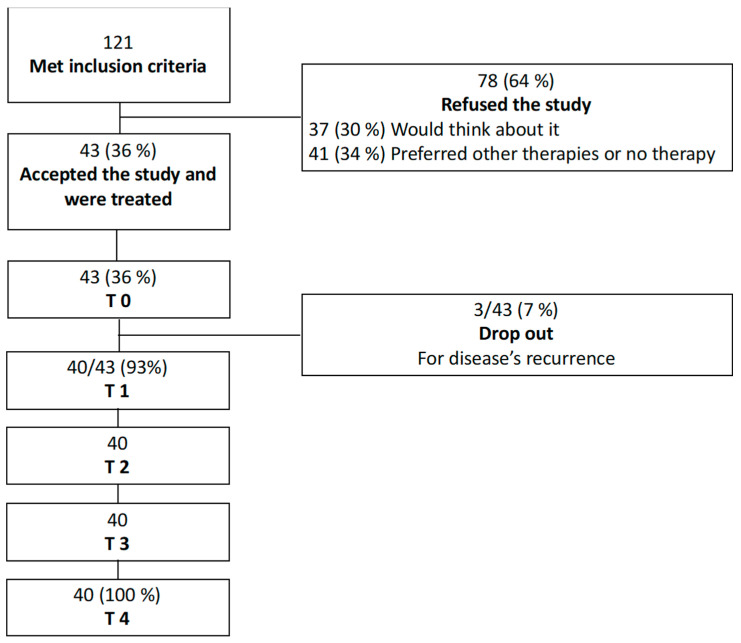
This figure reports the flow chart of the study. T0: baseline and first treatment, T1: first follow-up and second treatment, T2: second follow-up and third treatment, T3: third follow-up, T4: follow-up 6 months after the third treatment.

All scores and parameters, VHI, vaginal length and FSFI score, were recorded before each treatment, at baseline (T0) before the first treatment session, before second and third session (T1 and T2) and one month (T3) and six months after the last treatment (T4) (Figure 2). Patients that did not complete all the laser sessions, all questionnaires and physical evaluations, were excluded from the final analysis.

### 2.4. Statistical Analysis

Statistical analysis was performed using IBM SPSS Statistics 25.0 software (IBM Corp., Armonk, NY, USA). Continuous variables are presented as mean and standard deviation. Categorical data are expressed as percentages and absolute rates. Statistical comparisons of response rates were performed using contingency tables and the Pearson chi-squared test. Comparisons between baseline and follow-up values were performed by a two-tailed coupled *t*-test. *p* value was set to 0.05 for statistical significance. In order to test the influence of covariates for the distribution of vaginal length over time, in the follow-up period, the multivariable model with mixed effects for repeated measures was applied. As a result of this, the coefficient and standard error for each covariate in the model was calculated, together with the relative probability level (*p* value). Positive coefficients indicate a direct proportional association to increased vaginal length while negative coefficients indicate an inverse proportional correlation to increased vaginal length.

## 3. Results

### 3.1. Population and Oncological Data

We offered laser therapy to 121 women followed up in our outpatient clinic and that met the inclusion criteria. Forty-three (36%) women accepted and were treated, 37 (30%) replied that they “would think about it” and 41 (34%) that they preferred other therapies or no therapy.

The characteristics of the study population are shown in Table 1.

Tumor types included endometrial and cervical carcinoma mainly in the advanced stage. All patients received RT. Characteristics and doses of all previous treatments are shown in Table 2.

Forty out of forty-three women (93%) underwent the three planned sessions of laser therapy and completed all scheduled check-up. Three patients were diagnosed with metastatic disease shortly after the first laser application for vaginal symptoms, three to five years after primary cancer treatment. The vaginal complaints were not related to the symptoms from cancer recurrence.

### 3.2. Vaginal and Sexual Parameters

**Figure 3:**
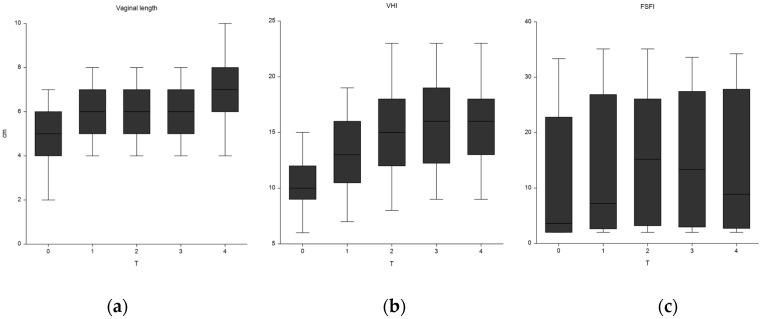
Clinical and functional improvement during treatment and follow-up of: (**a**) vaginal length, (**b**) vaginal health index (VHI), (**c**) female sexual function index (FSFI).

As shown in Table 3 and Figure 3, the vaginal parameters at T0 were characterized by severe atrophy and fibrosis. Moreover, a FSFI score below the normal range was recorded in all 40 patients.

All patients underwent the first treatment, but as mentioned above, three of them went off study before T1 due to a metastasis occurrence diagnosed shortly after the first laser treatment. The remaining 40 patients (93%) completed the planned treatments and the two follow-up visits after the last treatment. The mean time (± SD) elapsed between the three treatments was 45 ± 19 days (T0–T1), 44 ± 16 days (T1–T2) and 38 ± 10 days (T2–T3), respectively. T4 was performed 177 ± 56 days (mean ± SD) from the last laser therapy.

The vaginal length increased progressively and significantly after each session compared to baseline. The increase was 9% (*p* = 0.03) at T2 and 28% (*p* < 0.0001) at T3 (Table 3 and Figure 3). These positive results were maintained at T4 (*p* < 0.0001). VHI showed a significant improvement already after the first application (*p* < 0.0001). It progressively increased after each application until reaching a 57% improvement at T3. These results were confirmed at T4 (*p* < 0.0001).

The FSFI showed a 36% increase at T3 compared to T0, but this difference was not statistically significant and normal values (>26.5) were not achieved.

A correlation analysis revealed a significant trend toward improvement of both vaginal length (*r* = 0.3600, *p* < 0.0001) and VHI (*r* = 0.4897, *p* < 0.0001).

Regarding the improvement of the vaginal length, the multivariable analysis highlighted some unfavorable prognostic factors including: younger age at treatment, higher dose of RT and longer time since RT, lower BMI, previous cesarean section and cervical cancer (Table 4).

All procedures were well tolerated (VAS ranged between 1 and 3) without serious adverse effects, with only one patient reporting spotting after the first procedure and another patient reporting cystitis after the second procedure. No topical treatment was administered.

The patients that completed all laser sessions were asked if they would be willing to repeat the all procedure. Thirty-nine patients (97%) replied that they had benefited from the treatment and were available to repeat it while one patient declared that she did not report advantage, but that she would have repeated it anyway.

All patients were alive and disease free at the time of writing.

## 4. Discussion

Radiotherapy for cancer of the uterine cervix and endometrium always involves radiation of the vagina, causing severe long-term side effects. We found that CO_2_ laser therapy resulted in lengthening and functional improvement of the vagina. However, it did not improve the patient reported sexual health function. The procedure was well tolerated, the patients experienced minimal discomfort and no adverse effects were reported. To our knowledge, this is the first study evaluating the effects of laser therapy in this setting.

Vaginal changes after RT represent one of the major problems in the survivors after uterine carcinoma, especially in cervical cancers because of the young age of onset [7,37].Vaginal shortening and fibrosis impact on the quality of life and on the possibility of carrying out adequate oncological surveillance [23].

These structural changes of the vaginal wall have been confirmed by histological studies although data on this topic is reported with an extremely variable incidence ranging from 1.2% to 88% [38].

As reported in the available literature, laser therapy based on a non-ablative photodermal effect, seems to stimulate angiogenesis, fibroblast activity and new collagen formation without ablative and thermal damage [28]. Although the exact mechanism of how this type of treatment works is still unknown [27], several studies showed the benefit of laser treatment in menopausal genitourinary syndromes and breast cancer survivors [20]. Unfortunately, these studies included only a small number of patients, did not report on side effects and long-term results and were performed with different types of lasers (CO_2_, erbium) [24]. However, a systematic review described a rapid and statistically significant benefit on vaginal tissues with improved self-reported outcomes and clinical parameters [20].

In the present study, we analyzed a group of patients difficult to treat because the RT-induced damage is much more extensive compared to the effects of hypoestrogenism. Despite this, we have collected results similar to those reported in the latter setting. Notably, some improvement was observed already after the first session (Figure 3), with further improvements after each treatment, for at least six months as recorded at the clinical control (T4) for both parameters. More specifically, at the third laser application the parameters of vaginal atrophy (VHI > 15) and vaginal length (= 7 cm) were in the normal range. Furthermore, it is important to underline that the treatment was also well tolerated from a subjective point of view, as suggested by the excellent compliance (100% of patients who started laser therapy completed the three scheduled sessions) and by the declared willingness to repeat this treatment, if necessary, by of all patients.

Many authors [11] have tried to identify negative prognostic factors for vaginal stenosis and fibrosis in patients treated with RT such as type of tumor (cervical vs. endometrial), stage of the disease, type of treatment, radiation dose and age of the patient. The results are discordant and many of these studies are dated and have many biases [11]. The same factors were considered in our study in search of unfavorable indicators of response to laser therapy. As regards VHI, no specific predictive factor emerged from the multivariate analysis. Therefore, the improvement seems to be independent by the patients’ characteristics, the type of tumor and the delivered therapies. For vaginal length, the negative predictive factors were correlated with the severity of hypoestrogenism (younger age at diagnosis and BMI) and severity of vaginal fibrosis (correlated with time interval from RT, type of RT and doses). The study of Brand et al. [11] reported increased post-RT fibrosis after the age of 50, while Hartman and Diddle [39] reported age-independent RT effects on the vagina without correlation with BMI. Our results seem to suggest a negative correlation of the response to therapy with younger age probably due to more severe fibrosis and estrogen deprivation leading to a more resistant fibrosis.

We found that laser treatment was less effective in cervical cancers than endometrial tumors and that the resulting efficacy rate was affected by the radiation therapy doses. This confirms the literature data showing that vaginal toxicity is dose-dependent and more severe in cervical cancers [40]. However, we have not been able to measure a difference in response to BT and ERT as almost all our patients underwent both treatments.

The laser treatment program includes three cycles 30–40 days apart as attack therapy, with one cycle per year as maintenance therapy, although data on duration and frequency of maintenance therapy are discordant. Gambacciani et al. [41] reported positive results for at least 12 months while Pierrali et al. [42] observed a decline in patient satisfaction, from 76% to 52%, 3–25 months after the last procedure. This implies that the effect of the procedure is time-dependent and should be repeated. Our study shows the same positive results achieved at the third control (T3) even six months after (T4) the last treatment. This could suggest that the standard schedule of one laser session per year may also be favorably applied in RT-treated patients. Nonetheless, randomized studies with vaginal dilators should be performed in order to confirm our results.

In this study, we propose vaginal laser treatment as a possible therapeutic option for vaginal fibrosis after pelvic RT. The alternative treatments currently available include creams, gels, moisturizers with or without hormones and the use of vaginal dilators [28]. High response rates after local hormonal treatments in case of hypoestrogenism have been reported in the literature. However, patients undergoing pelvic RT develop radiation-induced reduction of vaginal hormone receptors [23].

Dilation during or immediately after RT can produce tissue damages and there is no evidence on prevention of stenosis. Therefore, this procedure should be used only after the resolution of the inflammatory phase [43]. Furthermore, some studies questioned the effectiveness of vaginal dilation and highlighted the low patients compliance (5–14%), probably due to long treatment times to achieve substantial results [17].

One of the limitations of the present study is that the effects of laser treatment have been evaluated only from a clinical point of view without performing biopsies to assess the histological changes. However, we avoided to perform biopsies due to the potential risk of fistulas. Another bias of the study may be originated from the selection of patients, because probably only the most motivated patients were treated. Finally, a better assessment of symptoms after the laser therapy and a longer follow-up are necessary in order to observe if the fibrosis and vaginal stenosis worsen over years and to set the necessity of retreat patients.

## 5. Conclusions

This promising, although preliminary, experience in the use of laser therapy in patients following pelvic RT put the focus on the need address the issue of sexual rehabilitation in cancer survivors. Conversely, some studies reported lack of education and attention on this topic. Further randomized trials are needed to confirm the results of laser treatment in this setting, to compare different therapeutic options and to evaluate combination of different strategies for patients with sexual impairment after pelvic RT.

## Figures and Tables

**Table 1:** Population and tumor characteristics of 43 patients treated.

Characteristics	Variable	Data
Time from the end of RT to laser treatment (months)	mean ± SD (range)	34 ± 29 (3–144)
Age at tumor diagnosis (years)	mean ± SD (range)	48 ± 8 (31–62)
Age at laser treatment (years)	mean ± SD (range)	51 ± 8 (34–64)
*Vaginal symptoms at baseline*		
Vaginal stenosis	N (%)	14 (33)
Dyspareunia	N (%)	6 (14)
Vaginal dryness	N (%)	12 (28)
Vaginal atrophy	N (%)	15 (35)
*BMI kg/m2*	mean ± SD	23 ± 3
*Previous pregnancy*	N (%)	32 (74)
*Modality of delivery*		
Cesarean section	N (%)	2 (4)
Natural delivery	N (%)	30 (70)
*Menopause*		
Yes	N (%)	42 (98)
No	N (%)	1 (2)
Iatrogenic	N (%)	22 (53)
Age at menopause (years)	mean ± SD (range)	46 ± 5 (31–55)
*Type of cancer*		
*Cervical*	N (%)	31 (72)
Early	N (%)	5 (12)
Locally advanced	N (%)	26 (60)
*Endometrial*	N (%)	12 (28)
Early stage	N (%)	9 (21)
Late stage	N (%)	3 (7)

BMI: body mass index, RT: radiotherapy, SD: standard deviation, N: number.

**Table 2:** Characteristics and doses of the treatments received by the study population.

Parameters	Variables	LG
Previous therapies		
EBRT+BT+CHT	N (%)	22 (51)
EBRT+BT+CHT+S	N (%)	10 (23)
EBRT+BT+S	N (%)	3 (7)
EBRT+CHT+S	N (%)	3 (7)
EBRT+CHT	N (%)	2 (5)
EBRT+S	N (%)	1 (2)
BT+S	N (%)	2 (5)
*EBRT*		
Dose (Gy)	mean ± SD	46 ± 1
Technique		
3D CRT	N (%)	24 (59)
IMRT	N (%)	17 (41)
Boost dose (Gy)	mean ± SD	12 ± 4
EBRT + boost dose (Gy)	mean ± SD	48 ± 5
*BT*		
Dose (Gy)	mean + SD	23 ± 10
Technique		
PDR	N (%)	4 (11)
HDR	N (%)	33 (89)

LG: laser group, EBRT: external radiotherapy, BT: brachytherapy, CHT: chemotherapy, S: surgery, N: number, Gy: Gray, 3D CRT: 3D conformal radiotherapy, IMRT: intensity, modulated radiotherapy, SD: standard deviation, PDR: pulsed dose rate, HDR: high dose rate.

**Table 3:** Vaginal parameters.

Time	Patients	VHI		Vaginal Length (cm)		FSFI	
T	*N*	mean ± SD	*p*	mean ± SD	*p*	mean ± SD	*p*
T0	43	10.4 ± 2.4		5.3 ± 1.2		11 ± 12	
T1	40	13.3 ± 3.1	<0.0001	5.7 ± 1.2	0.0962	14 ± 12	0.2808
T2	40	14.9 ± 3.5	<0.0001	5.8 ± 1.2	0.0313	15 ± 12	0.1298
T3	40	15.9 ± 3.8	<0.0001	6.8 ± 1.5	<0.0001	15 ± 12	0.1148
T4	40	15.7 ± 3.6	<0.0001	6.9 ± 1.4	<0.0001	15 ± 12	0.2257

N: patients number, T: time, T0: baseline and first treatment, T1: first follow-up and second treatment, T2: second follow-up and third treatment, T3: third follow-up, T4: fourth follow-up 6 months after third treatment. VHI: vaginal health index, cm: centimeters, FSFI: female sexual function index. SD: standard deviation, *p* values were calculated at each follow-up versus baseline values.

**Table 4:** Multivariable analysis. Variables associated with vaginal lengthening after laser therapy.

Variable	Coefficient	Standard Error	*p* < 0.05
Age at laser therapy	0.0035	0.0149	0.0000
Days since radiotherapy	−0.0133	0.0025	0.0058
BMI (kg/m^2^)	0.0122	0.0334	0.0000
History of a cesarean section (yes or no)	−14.987	0.3433	0.0120
History of EBRT (yes or no)	−0.3251	0.5167	0.0000
Cervical cancer (versus endometrial cancer)	0.4415	0.1427	0.0364

BMI: body mass index, EBRT: external radiotherapy.

## Supplementary Materials

The following are available online at https://www.mdpi.com/2072-6694/12/6/1639/s1, Table S1: Vaginal Health Index (VHI) in Original Version, Table S2: Vaginal Health Index (VHI) Italian Translation, Table S3: The Female Sexual Function Index (FSFI), Original Version, Table S4: The Female Sexual Function Index (FSFI), Italian Translation.
