# Supplementary material for: Results of a Phase I-II Study on Laser Therapy for Vaginal Side Effects after Radiotherapy for Cancer of Uterine Cervix or Endometrium
Source: Cancers (Basel). 2020 Jun 21;12(6):1639. doi: 10.3390/cancers12061639 (PMC7352893; doi:10.3390/cancers12061639)
Supplement: Supplementary file 1 [file cancers-12-01639-s001.pdf]

Supplementary Materials

Table S1. Vaginal Health Index (VHI) in Original Version.

Vaginal Health Index (VHI), Original Version					
Score	1	2	3	4	5
Elasticity	None	Poor	Fair	Good	Excellent
Fluid Volume (Pooling of secretion)	None	Scant amount	Superficial amount	Moderate amount of dryness	Normal amount
pH	≥ 6.1	5.6-6.0	5.1-5.5	4.7-5.0	≤ 4.6
Epithelial integrity	Petechiae noted before contact	Bleeds with light contact	Bleeds with scraping	Not friable- thin epithelium	normal
Moisture (coating)	None, surface inflamed	None, surface not inflamed	Minimal	Moderate	Normal

Table S2. Vaginal Health Index (VHI) Italian Translation

Vaginal Health Index (VHI), Italian Translation					
Punteggio	1	2	3	4	5
Elasticità complessiva	Nessuna	Scarsa	Discreta	Buona	Eccellente
Secrezione	Nessuna	Scarsa	Superficiale	Moderata	Normale
pH	≥ 6.1	5.6-6.0	5.1-5.5	4.7-5.0	≤ 4.6
Mucosa epiteliale	Petecchie presenti prima del contatto	Sanguina al contatto lieve	Sanguina allo sfregamento	Non friabile, mucosa sottile	Non friabile, mucosa normale
Idratazione	Assente, mucosa infiammata	Assente, mucosa non infiammata	Minima	Moderata	Normale

Table S3. The Female Sexual Function Index (FSFI), Original Version.

Q1: Over the past 4 weeks, how often did you feel sexual desire or interest?

5 = Almost always or always
4 = Most times (more than half the time)
3 = Sometimes (about half the time)
2 = A few times (less than half the time)
1 = Almost never or never

Q2: Over the past 4 weeks, how would you rate your level (degree) of sexual desire or interest?

5 = Very high
4 = High
3 = Moderate
2 = Low
1 = Very low or none at all

Q3: Over the past 4 weeks, how often did you feel sexually aroused ("turned on") during sexual activity or intercourse?

0 = No sexual activity
5 = Almost always or always
4 = Most times (more than half the time)
3 = Sometimes (about half the time)
2 = A few times (less than half the time)
1 = Almost never or never

Q4: Over the past 4 weeks, how would you rate your level of sexual arousal ("turn on") during sexual activity or intercourse?

0 = No sexual activity
5 = Very high
4 = High
3 = Moderate
2 = Low
1 = Very low or none at all

Q5: Over the past 4 weeks, how confident were you about becoming sexually aroused during sexual activity or intercourse?

0 = No sexual activity
5 = Very high confidence
4 = High confidence
3 = Moderate confidence
2 = Low confidence
1 = Very low or no confidence

Q6: Over the past 4 weeks, how often have you been satisfied with your arousal (excitement) during sexual activity or intercourse?

0 = No sexual activity
5 = Almost always or always
4 = Most times (more than half the time)
3 = Sometimes (about half the time)
2 = A few times (less than half the time)
1 = Almost never or never

Q7: Over the past 4 weeks, how often did you become lubricated ("wet") during sexual activity or intercourse?

0 = No sexual activity
5 = Almost always or always
4 = Most times (more than half the time)
3 = Sometimes (about half the time)
2 = A few times (less than half the time)
1 = Almost never or never

Q8: Over the past 4 weeks, how difficult was it to become lubricated ("wet") during sexual activity or intercourse

0 = No sexual activity
1 = Extremely difficult or impossible
2 = Very difficult
3 = Difficult
4 = Slightly difficult
5 = Not difficult

Q9: Over the past 4 weeks, how often did you maintain your lubrication ("wetness") until completion of sexual activity or intercourse?

0 = No sexual activity
5 = Almost always or always
4 = Most times (more than half the time)
3 = Sometimes (about half the time)
2 = A few times (less than half the time)

1 = Almost never or never

Q10: Over the past 4 weeks, how difficult was it to maintain your lubrication ("wetness") until completion of sexual activity or inter- course?

0 = No sexual activity
1 = Extremely difficult or impossible
2 = Very difficult
3 = Difficult
4 = Slightly difficult
5 = Not difficult

Q11: Over the past 4 weeks, when you had sexual stimulation or intercourse, how often did you reach orgasm (climax)?

0 = No sexual activity
5 = Almost always or always
4 = Most times (more than half the time)
3 = Sometimes (about half the time)
2 = A few times (less than half the time)
1 = Almost never or never

Q12: Over the past 4 weeks, when you had sexual stimulation or intercourse, how difficult was it for you to reach orgasm (climax)?

0 = No sexual activity
1 = Extremely difficult or impossible
2 = Very difficult
3 = Difficult
4 = Slightly difficult
5 = Not difficult

Q13: Over the past 4 weeks, how satisfied were you with your ability to reach orgasm (climax) during sexual activity or intercourse?

0 = No sexual activity
5 = Very satisfied
4 = Moderately satisfied
3 = About equally satisfied and dissatisfied
2 = Moderately dissatisfied
1 = Very dissatisfied

Q14: Over the past 4 weeks, how satisfied have you been with the amount of emotional closeness during sexual activity between you and your partner?

0 = No sexual activity
5 = Very satisfied
4 = Moderately satisfied
3 = About equally satisfied and dissatisfied
2 = Moderately dissatisfied
1 = Very dissatisfied

Q15: Over the past 4 weeks, how satisfied have you been with your sexual relationship with your partner?

5 = Very satisfied
4 = Moderately satisfied
3 = About equally satisfied and dissatisfied

2 = Moderately dissatisfied
1 = Very dissatisfied

Q16: Over the past 4 weeks, how satisfied have you been with your overall sexual life?

5 = Very satisfied
4 = Moderately satisfied
3 = About equally satisfied and dissatisfied
2 = Moderately dissatisfied
1 = Very dissatisfied

Q17: Over the past 4 weeks, how often did you experience discomfort or pain during vaginal penetration?

0 = Did not attempt intercourse
1 = Almost always or always
2 = Most times (more than half the time)
3 = Sometimes (about half the time)
4 = A few times (less than half the time)
5 = Almost never or never

Q18: Over the past 4 weeks, how often did you experience discomfort or pain following vaginal penetration?

0 = Did not attempt intercourse
1 = Almost always or always
2 = Most times (more than half the time)
3 = Sometimes (about half the time)
4 = A few times (less than half the time)
5 = Almost never or never

Q19: Over the past 4 weeks, how would you rate your level (degree) of discomfort or pain during or following vaginal penetration?

0 = Did not attempt intercourse
1 = Very high
2 = High
3 = Moderate
4 = Low
5 = Very low or none at all

Domain	Questions	Score Range	Factor	Minimum Score	Maximum Score
Desire	1, 2	1–5	0.6	1.2	6.0
Arousal	3, 4, 5, 6	0–5	0.3	0	6.0
Lubrication	7, 8, 9, 10	0–5	0.3	0	6.0
Orgasm	11, 12, 13	0–5	0.4	0	6.0
Satisfaction	14, 15, 16	0–5	0.4	0	6.0
Pain	17, 18, 19	0–5	0.4	0	6.0

Full score range: 2.0–36.0.

Table S4. The Female Sexual Function Index (FSFI), Italian Translation.

D1: Nelle passate 4 settimane con quale frequenza hai provato interesse o desiderio sessuale?

5 = Quasi sempre o sempre

4 = Il più delle volte (più della metà)
3 = Qualche volta (circa la metà)
2 = Poche volte (meno della metà)
1 = Quasi mai o mai

D2: Nelle passate 4 settimane, come definiresti il tuo livello di interesse o desiderio sessuale?

5 = Molto elevato
4 = Elevato
3 = Moderato
2 = Basso
1 = Molto basso o nullo

D3: Nelle passate 4 settimane, quanto spesso ti sei sentita sessualmente eccitata durante le attività sessuali o durante i rapporti sessuali?

0 = Nessuna attività sessuale
5 = Quasi sempre o sempre
4 = Il più delle volte (più della metà)
3 = Qualche volta (circa la metà)
2 = Poche volte (meno della metà)
1 = Quasi mai o mai

D4: Nelle passate 4 settimane, come quantificheresti il tuo livello di eccitamento sessuale durante le attività sessuali o nei rapporti completi?

0 = Nessuna attività sessuale
5 = Molto elevato
4 = Elevato
3 = Moderato
2 = Basso
1 = Molto basso o nullo

D5: Nelle passate 4 settimane, quanto ti sentivi sicura di riuscire ad eccitarti durante le attività sessuali o nei rapporti sessuale?

0 = Nessuna attività sessuale
5 = Molto sicura
4 = Sicura
3 = Moderatamente sicura
2 = Poco sicura
1 = Molto poco o per niente sicura

D6: Nelle passate 4 settimane, con quale frequenza ti sei sentita soddisfatta della tua eccitazione durante le attività sessuali o nei rapporti completi?

0 = Nessuna attività sessuale
5 = Quasi sempre o sempre
4 = Il più delle volte (più della metà)
3 = Qualche volta (circa la metà)
2 = Poche volte (meno della metà)
1 = Quasi mai o mai

D7: Nelle passate 4 settimane, quante volte ti sei sentita lubrificata (bagnata) durante le attività sessuali o nei rapporti completi?

0 = Nessuna attività sessuale
5 = Quasi sempre o sempre

4 = Il più delle volte (più della metà)
3 = Qualche volta (circa la metà)
2 = Poche volte (meno della metà)
1 = Quasi mai o mai

D8: Nelle passate 4 settimane, quanto è stato difficoltoso diventare lubrificata (bagnata) durante le attività sessuali o nei rapporti completi?

0 = Nessuna attività sessuale
5 = Estremamente difficile o impossibile
4 = Molto difficile
3 = Difficile
2 = Poco difficile
1 = Non difficile

D9: Nelle passate 4 settimane, quanto spesso sei riuscita a mantenere la lubrificazione vaginale fino alla fine delle attività sessuali o dei rapporti completi?

0 = Nessuna attività sessuale
5 = Quasi sempre o sempre
4 = Il più delle volte (più della metà)
3 = Qualche volta (circa la metà)
2 = Poche volte (meno della metà)
1 = Quasi mai o mai

D10: Nelle passate 4 settimane, quanto è stato difficile mantenere la lubrificazione vaginale fino alla fine delle attività sessuali o dei rapporti completi?

0 = Nessuna attività sessuale
5 = Estremamente difficile o impossibile
4 = Molto difficile
3 = Difficile
2 = Poco difficile
1 = Non difficile

D11: Nelle passate 4 settimane, durante i rapporti completi o le stimolazioni sessuali, quanto spesso hai raggiunto l'orgasmo?

0 = Nessuna attività sessuale
5 = Quasi sempre o sempre
4 = Il più delle volte (più della metà)
3 = Qualche volta (circa la metà)
2 = Poche volte (meno della metà)
1 = Quasi mai o mai

D12: Nelle passate 4 settimane, durante i rapporti completi o le stimolazioni sessuali, quanto è stato difficile raggiungere l'orgasmo?

0 = Nessuna attività sessuale
1 = Estremamente difficile o impossibile
2 = Molto difficile
3 = Difficile
4 = Poco difficile
5 = Non difficile

D13: Nelle passate 4 settimane, quanto ti ha soddisfatta la tua capacità di raggiungere l'orgasmo durante i rapporti completi o le stimolazioni sessuali?

0 = Nessuna attività sessuale
5 = Molto soddisfatta
4 = Moderatamente soddisfatta
3 = Soddisfatta e insoddisfatta nella stessa misura
2 = Moderatamente insoddisfatta
1 = Molto insoddisfatta

D14: Nelle passate 4 settimane, quanto ti ha soddisfatto l'affinità emotiva con il tuo partner durante le attività sessuali?

0 = Nessuna attività sessuale
5 = Molto soddisfatta
4 = Moderatamente soddisfatta
3 = Soddisfatta e insoddisfatta nella stessa misura
2 = Moderatamente insoddisfatta
1 = Molto insoddisfatta

D15: Nelle passate 4 settimane, quanto ti ha soddisfatto la relazione sessuale con il tuo partner?

5 = Molto soddisfatta
4 = Moderatamente soddisfatta
3 = Soddisfatta e insoddisfatta nella stessa misura
2 = Moderatamente insoddisfatta
1 = Molto insoddisfatta

D16: Nelle passate 4 settimane, quanto ti ha soddisfatto nel complesso la tua vita sessuale?

5 = Molto soddisfatta
4 = Moderatamente soddisfatta
3 = Soddisfatta e insoddisfatta nella stessa misura
2 = Moderatamente insoddisfatta
1 = Molto insoddisfatta

D17: Nelle passate 4 settimane, quanto spesso hai provato fastidio o dolore durante la penetrazione vaginale?

0 = Non ho provato ad avere rapporti completi
1 = Quasi sempre o sempre
2 = Il più delle volte (più della metà)
3 = Qualche volta (circa la metà)
4 = Poche volte (meno della metà)
5 = Quasi mai o mai

D18: Nelle passate 4 settimane, quanto spesso hai provato fastidio o dolore dopo la penetrazione vaginale?

0 = Non ho provato ad avere rapporti completi
1 = Quasi sempre o sempre
2 = Il più delle volte (più della metà)
3 = Qualche volta (circa la metà)
4 = Poche volte (meno della metà)
5 = Quasi mai o mai

D19: Nelle passate 4 settimane, come definiresti il tuo livello di fastidio o dolore durante o successivo alla penetrazione vaginale?

0 = Non ho provato ad avere rapporti completi
1 = Molto elevato
2 = Elevato
3 = Moderato
4 = Basso

5 = Molto basso o nullo

Dominio	Domanda	Range Di Punteggio	Fattore	Punteggio Minimo	Punteggio Massimo
Desiderio	1, 2	1–5	0.6	1.2	6.0
Eccitazione	3, 4, 5, 6	0–5	0.3	0	6.0
Lubrificazione	7, 8, 9, 10	0–5	0.3	0	6.0
Orgasmo	11, 12, 13	0–5	0.4	0	6.0
Soddisfazione	14, 15, 16	0–5	0.4	0	6.0
Dolore	17, 18, 19	0–5	0.4	0	6.0

Range di punteggio totale: 2.0–36.0.
